# Inhibition of catechol-*O*-methyltransferase by natural pentacyclic triterpenes: structure–activity relationships and kinetic mechanism

**DOI:** 10.1080/14756366.2021.1928112

**Published:** 2021-05-24

**Authors:** Fang-Yuan Wang, Gui-Lin Wei, Yu-Fan Fan, Dong-Fang Zhao, Ping Wang, Li-Wei Zou, Ling Yang

**Affiliations:** Institute of Interdisciplinary Integrative Medicine Research, Shanghai University of Traditional Chinese Medicine, Shanghai, China

**Keywords:** catechol-O-methyltransferase (COMT), pentacyclic triterpenes, enzymatic activity inhibition, mitochondrial membrane potential (MMP)

## Abstract

Inhibitors of COMT are clinically used for the treatment of Parkinson’s disease. Here, we report the first natural pentacyclic triterpenoid-type COMT inhibitors and their structure-activity relationships and inhibition mechanism. The most potent compounds were found to be oleanic acid, betulinic acid and celastrol with IC_50_ values of 3.89–5.07 μM, that acted as mixed (uncompetitive plus non-competitive) inhibitors of COMT, representing a new skeleton of COMT inhibitor. Molecular docking suggested that they can specifically recognise and bind with the unique hydrophobic residues surrounding the catechol pocket. Furthermore, oleanic acid and betulinic acid proved to be less disruptive of mitochondrial membrane potential (MMP) compared to tolcapone, thus reducing the risk of liver toxicity. These findings could be used to produce an ideal lead compound and to guide synthetic efforts in generating related derivatives for further preclinical testing.

## Graphical Abstract

**Figure:**
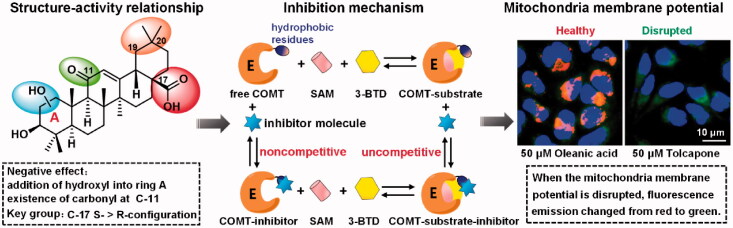

## 1. Introduction

Catechol-*O*-methyltransferase (COMT, C.E. 2.1.6) is a bisubstrate enzyme, that catalyses methyl transfer from S-adenosyl-L-methionine (SAM) to one of the hydroxyls of catecholamine neurotransmitters dopamine, epinephrine and norepinephrine, and catechol oestrogens, resulting in termination of their biological activity^1^^,^^2^. The catalytic site consisted of SAM and catechol binding pockets connected by a narrow channel through which methyl transfer occurs, where the SAM site proved to be deeply embedded^1^. COMT followed an ordered reaction mechanism where SAM bound first to the enzyme and the catechol substrate followed by release of the products in the reverse order^3^. COMT is a therapeutic target for the treatment of various peripheral cancers^4–6^ and central system disorders^7–10^. In particular, inhibition of peripheral COMT offers a unique advantage, since COMT inhibitors are clinically used as adjunct to levodopa (L-dopa) for the treatment of Parkinson’s disease^7^.

Marketed COMT inhibitors are nitrocatechols including tolcapone^11^, entacapone^12^ and opicapone^13^. These three drugs are co-administered with L-dopa, a precursor of dopamine, to increase its half-life and improve symptoms resulting from dopamine level fluctuation^14^^,^^15^. However, tolcapone was proposed to cause liver toxicity and thus required clinical liver function monitoring^15^. Although not related to idiosyncratic toxicity, entacapone can cause severe diarrhoea, increased dyskinesia frequency and has low bioavailability^16^. Therefore, the discovery of superior COMT inhibitors with better safety profiles and clinical efficacy is urgently needed.

To improve the complications of nitrocatechol drugs, researchers seek to discovery non-nitrocatechol COMT inhibitors from natural products, which may have the potential to improve toxicity profiles versus tolcapone and entacapone. There have been limited reports of natural COMT inhibitors. Notably, epigallocatechin and epicatechin (the major polyphenol in green tea)^17^, and chlorogenic acid and caffeic acid existent in coffee^18^ demonstrated COMT inhibition with the IC_50_ values of 6.3–60 μM. Some alkaloids and bioflavonoids (e.g. quercetin and fisetin) also showed inhibitory potency on COMT^19^^,^^20^. Unfortunately, the high polarity, acidity or alkalinity of these compounds makes it difficult to optimise them for use as drugs.

Pentacyclic triterpenes including four classes of oleananes, ursanes, lupanes and friedelanes are an excellent reservoir of biologically active compounds^21^. In the present study, we discovered natural COMT inhibitors with characterisation of a chemical scaffold of pentacyclic triterpene, which was distinct from previously reported compounds. An enzymatic activity based fluorescence assay was developed to evaluate COMT inhibitors with 3-BTD or a COMT-specific fluorescent probe as the substrate. That produced three potent inhibitors oleanic acid, betulinic acid and celastrol with IC_50_ values of 4.74, 5.07 and 3.89 μM, respectively. It seemed certain that introducing hydroxyl groups into the ring A of oleanic acid was not beneficial to COMT inhibition, while the C-17 site carboxyl of betulinic acid and the conjugated system between ring A and B of celastrol were necessary. These compounds inhibited COMT-catalysed *O*-methylation of 3-BTD in a mixed (uncompetitive and non-competitive) modality. Furthermore, docking simulation indicated that they can specifically bind with the unique hydrophobic residues (Met-40, Leu-198, Trp-143, Trp-38 and Pro-174) as a “gatekeeper” to guard the catechol site of COMT. Finally, oleanic acid and betulinic acid showed less potent toxicity on the normal human liver-derived cell line LO-2 and on mitochondrial membrane potential (MMP) compared to tolcapone, mitigating the risk of hepatotoxicity. These pentacyclic triterpenes represented a novel type of COMT inhibitors with improved safety profile, which might be further optimised to obtain related derivatives for preclinical testing.

## 2. Materials and methods

### 2.1. Chemicals and regents

Twenty natural pentacyclic triterpenes, quercetin and epicatechin were purchased from Chengdu Desite Biotechnology Co., Ltd. (Chengdu, China), and their purities more than 98%. Dithiothreitol (DTT, ≥99%), S-adenosyl-L-methionine (SAM, ≥80%), magnesium chloride hexahydrate (MgCl_2_^.^6H_2_O, ≥99%), entacapone (C_14_H_15_N_3_O_5_, ≥98%) and tolcapone (C_14_H_11_NO_5_, ≥98%) were supplied by Sigma-Aldrich Co. LLC. (Shanghai, China). 3-(Benzo[d]thiazol-2-yl)-7,8-dihydroxy-2H-chromen-2-one (3-BTD, ≥99%), 3-(Benzo[d]thiazol-2-yl)-7-dihydroxy-8-methoxy-2H-chromen-2-one (3-BTMD, ≥99%) and recombinant human S-COMT were made in our laboratory [22]. Phosphate buffer saline (50 mM, pH 7.4), Dulbecco's modified eagle medium (DMEM, with high glucose, pyruvate and L-glutamine), penicillin/streptomycin (PS, sterile), cell counting kit-8 (CCK-8), Hoechst 33342 and JC-10 dye were obtained from Dalian Meilun Biotechnology Co., Ltd. (Dalian, China). Foetal bovine serum (FBS, Gbico^®^) was purchase from Thermo Fisher Scientific Co., Ltd. (Shanghai, China). Acetonitrile (ACN, ≥99.9%), dimethyl sulfoxide (DMSO, ≥99.7%) and formic acid (CH_3_COOH, ≥98%) were of HPLC grade and purchased from Sigma-Aldrich Co. LLC. (Shanghai, China). Ultrapure water (18.2 MΩ cm) was produced using a Millipore water purification system (Milford, MA, U.S.A.).

### 2.2. COMT activity inhibition assay

The measurement of COMT activity was carried out as described with modification^22^. To determine IC_50_ values of COMT inhibition, the reaction was performed in the incubation mixture containing recombinant human S-COMT (2.0 μg/mL), MgCl_2_ (5 mM), DTT (1 mM), SAM (200 μM), 3-BTD (2 μM) and varying concentrations (from 0.125 to 20 μM) of tested compounds in a final volume of 200 μL PBS buffer (50 mM, pH 7.4). The tested compound of 20 mM stock in DMSO was used to prepare 10-point 2-fold dilution series. After pre-incubation 37 °C for 3 min, the reaction was initiated upon the addition of 10 μL SAM and progressed at 37 °C for 6 min. The incubation was terminated with 200 μL ice-cold ACN containing 1% formic acid. After centrifugation at 20,000 ×*g* for 5 min at 4 °C, 200 μL of supernatant was plated into assay wells (Costar^®^ assay plate, 96 well, black, flat bottom black, polystyrene plate, ref. #3925 from Corning Inc.). The fluorescent intensity were measured using the multi-mode microplate reader (SpectraMaxs iD3^®^, Molecular Devices, Austria). An additional assay include a DMSO treatment which served as a maximum enzyme activity control. The excitation/emission wavelengths set at 390/510 nm. Residual COMT activity can be calculated using the formula: (COMT inhibitor signal/DMSO signal) × 100%. To evaluate inhibition kinetics, the concentrations of 3-BTD substrate ranged from 0.01 to 5 μM in the presence of oleanic acid, celastrol and betulinic acid with the concentrations of 2.5, 5 and 7.5 μM. The fluorescence signal was detected as detailed above except the rate of *O*-methylation of 3-BTD which was expressed as nanomoles of methylated product formed per min per milligram of recombinant human S-COMT protein (nmol/min/mg protein).

### 2.3. Cytotoxicity assay

Cytotoxicity assays of oleanic acid, celastrol, betulinic acid, tolcapone and entacapone were performed using standard CCK8 kit. The normal human liver-derived cell line LO-2 were grown in DMEM culture medium supplemented with 10% FBS, penicillin (100 U/mL) and streptomycin (100 mg/mL). After culturing to 80% confluence, cells were digested and exactly 100 µL of cell suspension with a cell concentration of 10^4^ cells/well was seeded in 96-well plates and incubated in a 5% CO_2_ humidified atmosphere at 37 °C for 12 h. Then the cells were incubated with 100 μL tested compounds of varying concentrations from 0.25 to 100 μM prepared in FBS-free culture medium for 24 h. Subsequently, CCK-8 was diluted 1:10 with DMEM culture and them was added into the adherent cells and incubated in 37 °C for 2 h. The absorbance at 450 nm was measured by using the multi-mode microplate reader. Cell viability can be calculated by A/A_0_ × 100% (A and A_0_ are the absorbance of experimental group and control group, respectively).

### 2.4. Mitochondrial membrane potential assay

Toxic effects of oleanic acid, betulinic acid and tolcapone on mitochondrial membrane potential of LO-2 cells were examined. The cells were cultured as described in the Section 2.3. Then the cells were incubated with 100 μL tested compounds of various concentrations (from 0.25 to 25 μM prepared in FBS-free culture medium) for 24 h. To assess the mitochondria membrane potential, JC-10 (5 μg/mL) was incubated with the cells for 30 min, and Hochest (10 μg/mL) was used to stain nuclei. After removal of culture medium and rinsing three times with PBS, cell imaging and quantitative analysis were performed by high-content imaging analysis (Molecular Devices^®^ ImageXpress Micro 4, the USA).

### 2.5. Molecular docking

Molecular docking was carried out to determine geometrically and energetically stable conformation upon the binding of compounds to COMT. The protein structure of human S-COMT was obtained from a Protein Data Bank (PDB ID: 3BWM). All docking experiments were conducted through AutoDock Vina. To process the receptor file (.pdb) for docking, the following procedures were implemented: water molecules and irrelevant heteroatoms were removed, hydrogen atoms were added and non-polar hydrogens were subsequently merged, ultimately, charges were added using the Kollman method. The ligand compounds depicted in Chem3D were imported into ADT accompanied with the Torsion Tree root detected and were subsequently saved as .pdbqt files. To further verify its inhibition mechanism, the searching grid box was sufficiently large to wrap the whole protein. The conformational search space was generated with a spacing of 0.375 Å and dimensions of (126 × 122 × 106) points (.gpf file) with the grid centre XYZ coordinates at −9.82, −5.60 and −9.83, respectively. The highest ranked and lowest energy docking pose were further analysed by Discovery Studio visualiser version.

### 2.6. Data analyses

The kinetic analysis were performed by fitting the initial velocity as a function of concentration to the following equations using the non-linear regression analysis program in GraphPad Prism 5.0 software:

$$v=\frac{V_{\max}[S]}{{(K}_{m}+[S])(1+\frac{\left[I\right]}{K_{i}})}\mathrm{non}-\text{competitive inhibition}$$

$$v=\frac{V_{\max}[S]}{K_{m}+[S](1+\frac{\left[I\right]}{\alpha K_{i}})}\text{ uncompetitive inhibition}$$

$$v=\frac{V_{\max}[S]}{K_{m}(1+\frac{\left[I\right]}{K_{i}})+[S]}\text{ competitive inhibition}$$

$$v=\frac{V_{\max}[S]}{K_{m}(1+\frac{\left[I\right]}{K_{i}})+(1+\frac{\left[I\right]}{{\alpha K}_{i}})[S]}\text{ mix type inhibition}$$

The [S] and [I] represent the concentrations of substrate and inhibitor, respectively; *V_max_* is the maximal velocity; *K_m_* is the Michaelis constant; *K_i_* is the inhibition constant suggesting the dissociation of the enzyme-inhibitor complex (EI); α*K_i_* is the inhibition constant when the inhibitor binds to an enzyme-substrate complex, suggesting the dissociation constant of the enzyme-substrate-inhibitor complex (ESI); the value of α equals 1, meaning the pure non-competitive inhibition; at α ≫ 1, meaning competitive inhibition; at α ≪ 1, meaning uncompetitive inhibition. The reciprocals of velocity and substrate concentrations gave the linear correlation by which *K_m_* and *V_max_* values can be calculated. Obtained data were presented as mean ± standard error (± SE) of three independent experiments with duplicate determinations for each assay. Statistical difference in IC_50_ values were determined using an unpaired two-tailed *t*-test, which indicated as *p < .05, **p < .01, ***p < .001.

## 3. Results and discussion

### 3.1. Inhibition of COMT activity by pentacyclic triterpenes

**Figure 1.**
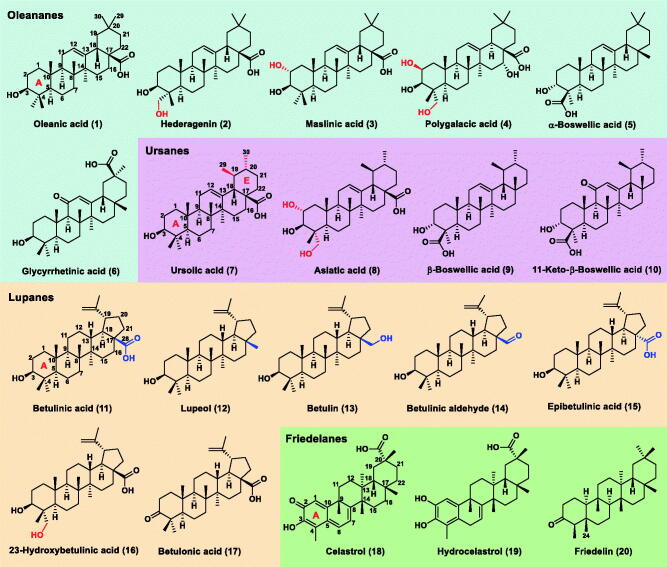
Chemical structures of tested twenty natural pentacyclic triterpenes. The oleanic acid, ursolic acid, betulinic acid, celastrol and their related analogues are numbered with compounds **1**–**6**, **7**–**10**, **11**–**17** and **18**–**20**, respectively.

**Figure 2.**
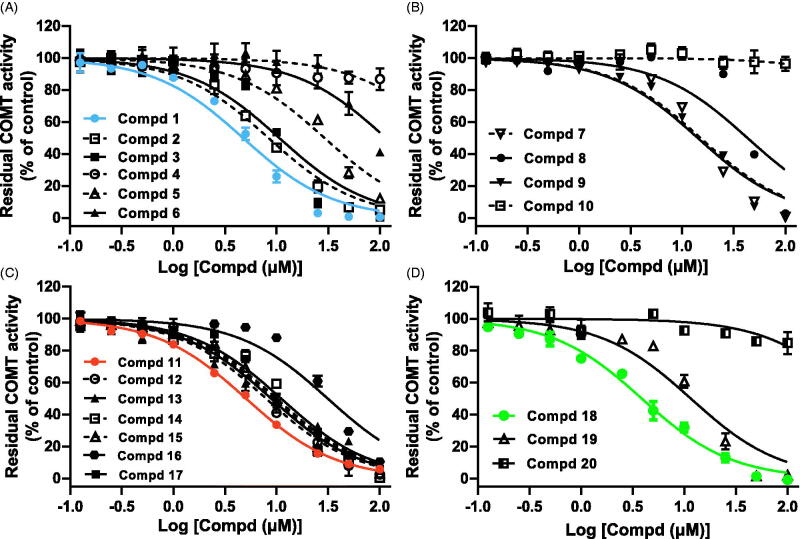
The concentration-dependent inhibition of human S-COMT-mediated *O*-methylation of 3-BTD with increasing concentrations (from 0.125 to 20 μM) of compounds **1** to **6** (A), **7** to **10** (B), **11** to **17** (C), and **18** to **20** (D). The error bars represent the standard deviation (±SD) of three independent experiments with duplicate determinations. These data were used to obtain IC_50_ values (Table 1).

To seek novel COMT inhibitors, an enzymatic activity-based fluorescent assay was established to define the ability of pentacyclic triterpenes to inhibit human S-COMT. The use of 3-BTD or a COMT-specific two-photon fluorescent probe^22^, greatly improve the sensitivity and reliability of this assay. Supplementary Scheme S1 illustrates the fluorescent responsive mechanism of the substrate 3-BTD that is catalysed by COMT. The optimised conditions for the incubation time and substrate concentration were given in Supplementary Figure S1. A series of natural pentacyclic triterpenes including oleananes (1–6), ursanes (7–10), lupanes (11–17) and friedelanes (18–20) were collected (Figure 1), and the inhibitory potency on COMT activity was evaluated. We plotted the concentration-dependent curves of these tested compounds with increasing concentrations from 0.125 to 200 μM (Figure 2), and the IC_50_ values ranged from 3.89 ± 0.15 to 115 ± 11.6 μM (Table 1). The most potent COMT inhibitors are oleanolic acid (**1**, 4.74 ± 0.29 μM), betulinic acid (**11**, 5.07 ± 0.087 μM) and celastrol (**18**, 3.89 ± 0.15 μM), comparable to the reported quercetin [20] (IC_50_=3.23 ± 0.11 μM) and epicatechin [17] (IC_50_=9.57 ± 0.55 μM). The potential pentacyclic triterpene-type COMT inhibitors have not been characterised previously.

**Table 1.** The IC_50_ values of pentacyclic triterpenes, quercetin and epicatechin versus recombinant human S-COMT as determined by using enzymatic activity based the fluorescence assay.

Compd	Chemical name	IC_50_ (μM)
**1**	Oleanic acid	4.74 ± 0.29*
**2**	Hederagenin	8.37 ± 0.46**
**3**	Masline acid	10.64 ± 0.96**
**4**	Polygalacic acid	N.E.
**5**	α-Boswellic acid	29.20 ± 1.65***
**6**	Glycyrrhetinic acid	114.90 ± 11.58***
**7**	Ursolic acid	15.13 ± 1.35**
**8**	Asiatic acid	43.11 ± 5.92***
**9**	β-Boswellic acid	14.21 ± 0.87***
**10**	11-Keto-β-Boswellic acid	N.E.
**11**	Betulinic acid	5.07 ± 0.087**
**12**	Lupeol	7.93 ± 0.47**
**13**	Betulin	8.86 ± 0.46**
**14**	Betulinic aldehyde	10.05 ± 0.63**
**15**	Epibetulinic acid	9.38 ± 0.37**
**16**	23-Hydroxybetulinic acid	31.42 ± 2.42***
**17**	Betulonic acid	11.11 ± 0.51**
**18**	Celastrol	3.89 ± 0.15
**19**	Hydrocelastrol	12.64 ± 0.73**
**20**	Friedelin	N.E.
	Quercetin	3.23 ± 0.11*
	Epicatechin	9.57 ± 0.55**

This inhibition assay was carried out with 2.0 μg/mL human S-COMT and 2.0 μM 3-BTD as the substrate under conditions described in Section 2.2. The IC_50_ value of each compound that significantly differed from that of celastrol (**18**) was determined using an unpaired two-tailed *t*-test as indicated by *p < 0.05, **p < 0.01 and ***p <0 .001. N.E. is the abbreviation of no effect. All the data were the mean ± standard error (± SE) of three independent experiments with duplicate determinations.

Of the oleanane-type triterpenes, oleanolic acid (1) displayed inhibitory potency on COMT activity in a concentration-dependent manner [the blue curve in Figure 2(A)]. However, with the introduction of the hydroxyl group into ring A (seeing Figure 1 for ring labeling), the COMT inhibitory potency decreased such as hederagenin (2) and maslinic acid (3). Polygalacic acid (4**)** with more hydroxyl groups almost did not inhibit COMT activity and the IC_50_ value was not calculated due to insufficient concentration-response relationship. Thus, the decrease of lipophilicity of ring A did not enhance COMT inhibition. In spite of minor difference in the ring E substitution position of C-29 and C-30 methyl groups, ursolic acid (**7**, IC_50_ = 15.13 ± 1.35 μM) displayed ∼3-fold lower inhibitory potency than oleanolic acid (1). Introducing hydroxyl into ring A of ursolic acid (7) can weaken COMT inhibition, as it did in the case of asiatic acid (**8,** IC_50_ = 43.11 ± 5.92 μM). Addition of a carbonyl to the C-11 position, such as 11-keto-β-boswellic acid (10), set up a complete loss of inhibitory potency.

Among these lupane-type triterpenes, betulinic acid (11) had strong inhibitory potency with the IC_50_ value of 5.07 ± 0.087 μM [the orange curve in Figure 2(C)], nearly equal to oleanolic acid (**1**, IC_50_=4.74 ± 0.29 μM). With addition of one hydroxyl group to ring A, 23-hydroxybetulinic acid (16) showed a reduced COMT-inhibitory effect. Compounds **12**–**15** also proved to be less potency versus betulinic acid (11), indicating that the converting of (*S*)-C-17 carboxyl group to methyl (12), hydroxymethyl (13), carbonyl (14) and (*R*)-C-17 carboxyl group (15) can lead to a slight decrease. Compared to betulinic acid (11), epibetulinic acid (15) showed a 50% reduction in COMT-inhibitory potential, despite having a little difference in the configuration of the C-17 substituent. Of the friedlane-type molecules, celastrol (**18,** IC_50_=3.89 ± 0.15 μM) showed a stronger COMT-inhibitory effect [the green curve in Figure 2(D)]. This might be attributed to the existence of a conjugation system between rings A and B, increasing the lipophilicity of ring A.

**Figure 3.**
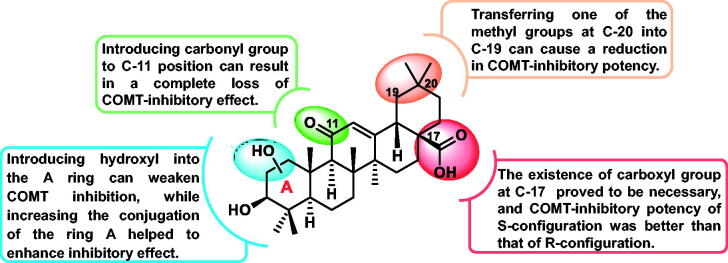
The structure-inhibition relationship of pentacyclic triterpenes against COMT activity.

Collectively, the primary structure-activity relationship (SAR) is summarised in Figure 3: (1) introducing hydroxyl groups into ring A weakened COMT inhibition, while increasing the conjugated system helped enhance the lipophilicity of ring A, which was beneficial for COMT inhibition; (2) the existence of a carbonyl group at the C-11 position resulted in a complete loss of inhibitory potency; (3) transferring one of the methyl groups at C-20 into C-19 can cause a reduction in COMT-inhibitory potency; (4) the C-17 carboxyl group was necessary, and the COMT inhibitory potential of S-configuration proved to be better than that of R-configuration. It was expected to guide us rational design and synthesis of novel COMT inhibitors of pentacyclic triterpene analogues based on the mentioned results.

### 3.2. Inhibition kinetics

**Figure 4.**
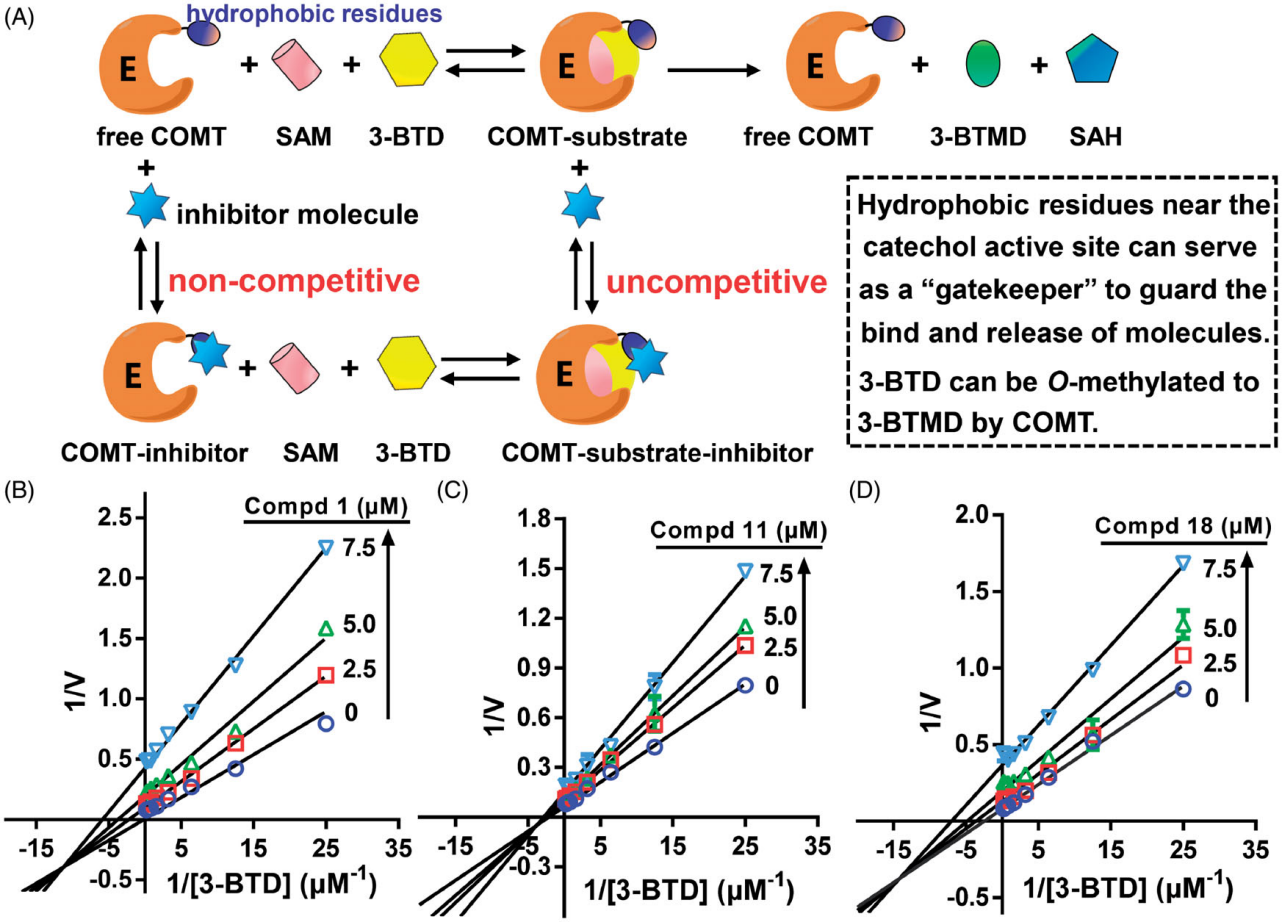
(A) Illustration of inhibition mechanism of the tested molecules for COMT-mediated *O*-methylation of 3-BTD. Lineweaver-Burk plots (transformed from Michaelis-Menten analysis) of compounds **1** (B), **11** (C) and **18** (D) of several concentrations were shown. The error bars represent the standard deviation (±SD) from three independent tests with duplicate determinations.

Figure 4(A) illustrated the inhibition mechanism of pentacyclic triterpenes for the catechol-binding site of COMT. Here, the probe 3-BTD, serving as a typical tool molecule to occupy the catechol pocket, can be *O*-methylated to 3-BTMD by COMT^22^. Instead of competing with 3-BTD, the inhibitor molecule can bind free COMT by recognising the unique hydrophobic residues surrounding the catechol site, and thus could act as a non-competitive inhibitior. In addition, the COMT-substrate complex bind with the inhibitor molecule which could hamper the release of the *O*-methylation product of 3-BTD. This was because the hydrophobic residues not only defined the catechol active site, but also served a “gatekeeper” to guard bind and release of molecules^14^. Thus, it was possible for the pentacyclic triterpene to inhibit the catechol site of COMT in a mixed (non-competitive plus uncompetitive) mode.

We monitored the kinetics in the steady-state as a function of 3-BTD in the presence of compounds **1**, **11** and **18** with saturated SAM (Supplementary Figure S3). The *O*-methylation rates of 3-BTD were plotted as Lineweaver-Burk diagrams [Figure 4(B–D)]. Obviously, the plot lines of Lineweaver-Burk were intersected in the quadrant III, suggesting that compounds **1**, **11** and **18** inhibited the catechol site in a non-competitive and uncompetitive manner. Furthermore, the *K_m_* and *V_max_* values for 3-BTD were determined to be 0.38 ± 0.029 μM and 13.8 ± 0.30 nmol/min/mg protein, respectively. With the addition of the inhibitors, the corresponding apparent *K_m_* and *V_max_* values significantly decreased. Thus, compounds **1**, **11** and **18** displayed a mixed (non-competitive plus uncompetitive) inhibition pattern with respect to the catechol active site of COMT. The global fit of the inhibition mode to these data yielded the *K_i_* values for the three ones in the range of 3.5–5.6 μM. Table 2 showed the kinetic parameters in detail.

**Table 2.** Kinetic parameters of inhibition of human S-COMT catalysed *O*-methylation of 3-BTD by compounds **1**, **11** and **18**

Compd	Con. (μM)	*K_m_* (μM)	*V_max_*(nmol/min/mg)	*K_i_* (μM)	α	Inhibition type	Goodness of fit (*R*^2^)
**1**	0	0.38 ± 0.029	13.85 ± 0.30	4.1 ± 0.30	0.29	Mixed	0.96
	2.5	0.30 ± 0.038***	8.45 ± 0.29***				
	5.0	0.19 ± 0.014***	4.56 ± 0.084***				
	7.5	0.15 ± 0.0088***	2.20 ± 0.030***				
**11**	0	0.38 ± 0.029	13.85 ± 0.30	5.6 ± 0.33	0.39	Mixed	0.98
	2.5	0.35 ± 0.025*	9.97 ± 0.20***				
	5.0	0.33 ± 0.030*	8.95 ± 0.22***				
	7.5	0.24 ± 0.038*	6.06 ± 0.24***				
**18**	0	0.40 ± 0.0078	13.12 ± 0.18	3.5 ± 0.51	0.20	Mixed	0.97
	2.5	0.22 ± 0.024***	7.91 ± 0.16***				
	5.0	0.12 ± 0.03***	4.39 ± 0.10***				
	7.5	0.11 ± 0.02***	2.54 ± 0.047***				

Kinetic assay was performed at 37 °C for 4 min using 2.0 μg/mL human S-COMT, 200 μM SAM, and various concentrations (from 0.02 to 5.0 μM) of 3-BTD. The values of *K_m_*, *V_max_*, *K_i_*, α and goodness of fit were calculated by the GraphPad Prism 5.0 software. Data were the mean ± standard error (± SE) of three independent experiments with duplicate measurements. Statistical difference in *K_m_* or *V_max_* values for each tested compound were determined using one-way ANOVA, which indicated as *p < 0.05, **p <0 .01, ***p < 0.001.

### 3.3. Molecular docking

**Figure 5.**
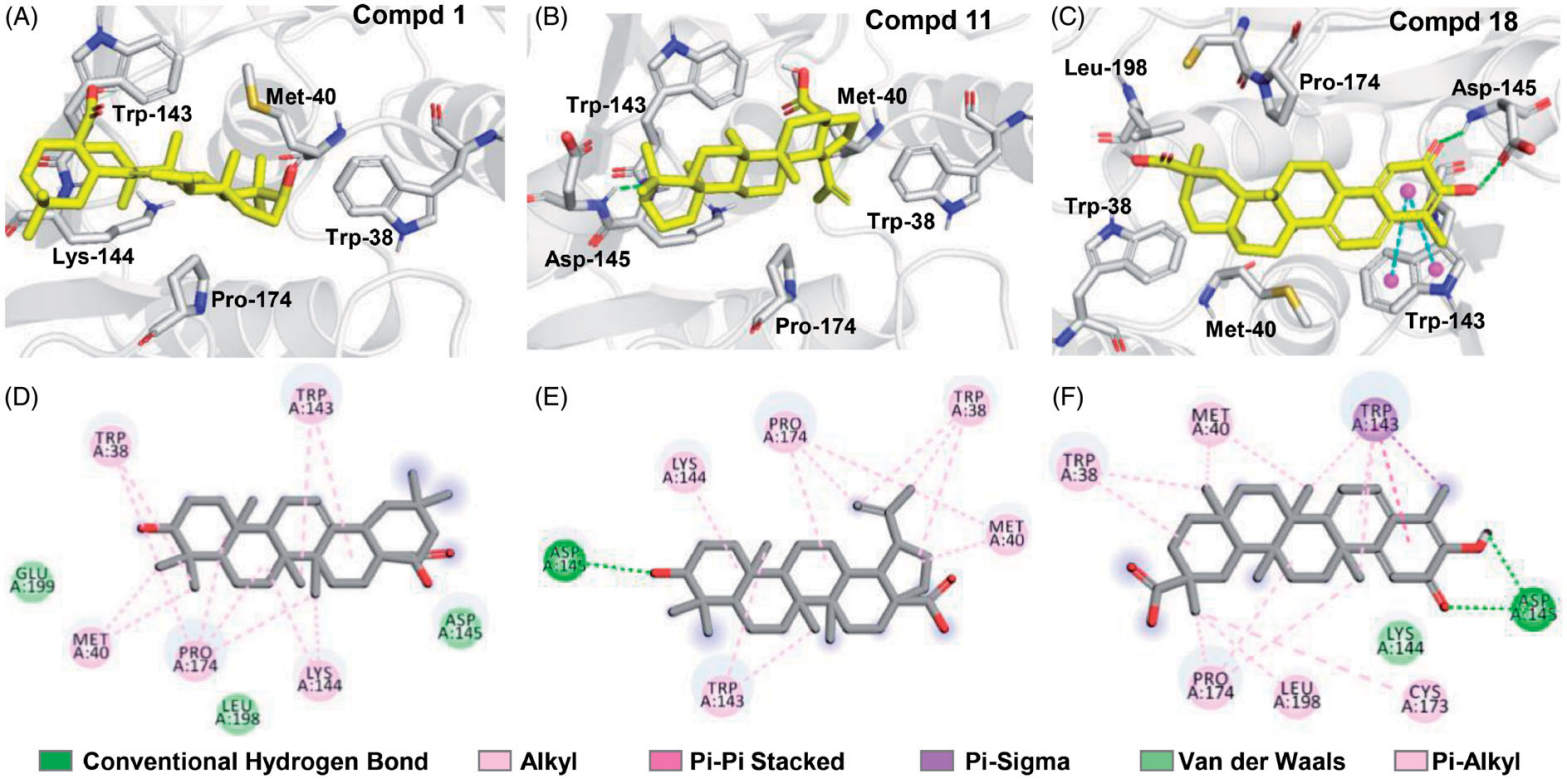
Molecular docking and the details for binding interactions of human S-COMT with compounds **1** (A and D), **11** (B and E) and **18** (C and F). The yellow molecule indicates the tested compound; the blue and red represent oxygen and nitrogen atoms, respectively; the dotted green and sky-blue lines depict hydrogen bond and π-π stacking interactions, respectively.

To further explore the binding interactions of compounds **1**, **11** and **18**, a three-dimensional model of human S-COMT (PDB: 3BWM) was constructed. The entirely hydrophobic residues (Met-40, Leu-98, Trp-143, Trp-38 and Pro-174) as “gatekeepers” surrounding the catalytic pocket of COMT were unique^14^^,^^23^^,^^24^. This allowed the specific recognition of some small molecules to COMT with hydrophobic interaction force. The three compounds covered the COMT active cave quite well and did not overlap with the catechol-binding pocket (Supplementary Figure S4). For compound **1**, the six-membered rings and some small hydrophobic substitutes can initiate alkyl and pi-alkyl interactions with Trp-38, Met-40, Pro-174, Trp-143 and the aliphatic portion of Lys-144 [seeing details in Figure 5(A and D)]. In the case of compound **11**, a key interaction involved a hydrogen bond between the negatively charged hydroxyl at the ring A and the side chain nitrogen of Asp-145 [Figure 5(B,E)]. For compound **18,** the binding interactions contained π-π packing between the phenyl moiety/the ring A and the indole ring of Trp-143 [sky blue dotted lines in Figure 5(C)], two hydrogen bonds between the carbonyl and hydroxyl groups of the ring A and the side chain of Asp-145 (green dotted lines) as well as alkyl and pi-alkyl interactions with Met-40, Trp-38, Pro-174, Leu-198 and Cys-173 [seeing the details in Figure 5(F)]. Obviously, the three molecules can be “trapped” by these “gatekeeper” residues around the active pocket, indicating binding with free COMT or COMT-substrate complex instead of binding the catechol- or SAM-pocket. What is more, the calculated binding energy (ΔE_binding_) values varied from −7.6 to −6.2 (Supplementary Table S1), revealing the following order of binding affinity to human S-COMT: compounds **18 **>** 1** > **11**. This is partially due to π-π interaction between ring A of compound **18** and the indole ring of Trp-143, which tightened its contact surface and the higher binding affinity with COMT. This was consistent with the biochemical data in which compound **18** had a lower *K_i_* value for inhibiting the *O*-methylation of 3-BTD.

### 3.4. Assessment of mitochondria membrane potential

**Figure 6.**
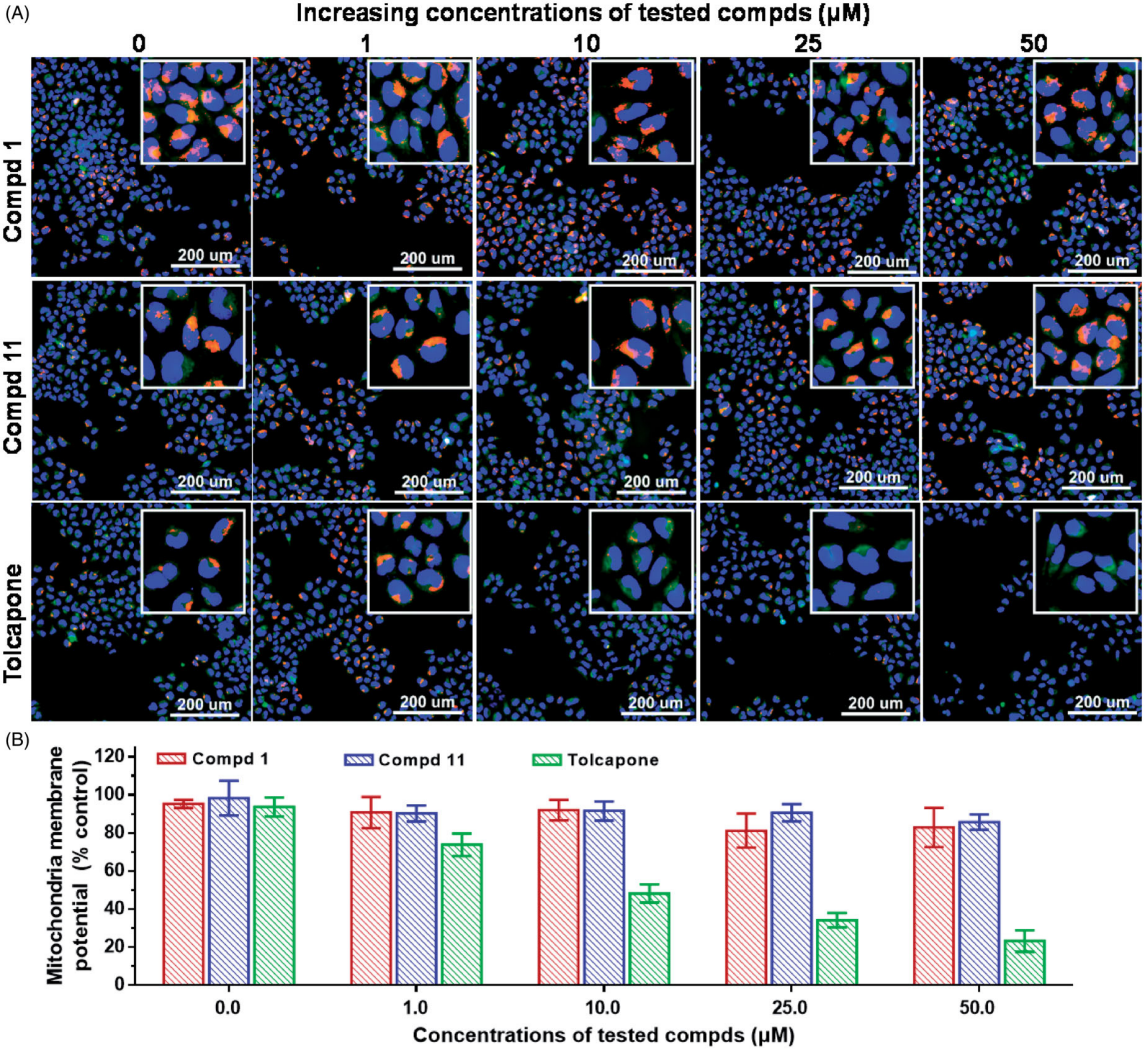
The effects of compounds **1**, **11**, and tolcapone on the mitochondrial membrane potential (MMP) of LO-2 cells. The cells were incubated with these compounds tested for 24 h, and then treated with JC-10 (5 μg/mL) and Hoechst (10 μg/mL) for 30 min. The red fluorescent images were collected at 575 nm excitation and 600–650 nm emission; the green images obtained at 475 nm excitation and 511–580 nm emission; the blue images collected at 405 nm excitation and 415–485 nm emission. The error bars represent standard deviation (± SD) from the results of three independent experiments with single determination for this assay.

To investigate the influence of the novel pentacyclic triterpene-type COMT inhibitors on hepatotoxicity, we examined the effects of these compounds on mitochondria membrane potential (MMP) using the normal human liver-derived cell line LO-2, along with tolcapone as a positive control. The dye JC-10 underwent a change in fluorescence emission from red to green when the MMP was disrupted and uncoupled^25^. For tolcapone, strong green signals were observed inside the cells with increasing concentrations [Figure 6(A), bottom row], indicating a decrease of MMP. In contrast, a strong red fluorescence appeared in the mitochondria with increasing concentrations of compounds **1** or **11** [Figure 6(A), top and middle rows]. This observation demonstrate that the two compounds tested had the lower mitochondria toxicity than tolcapone. In Figure 6(B), tolcapone at 10 μM led to a 50% reduction in membrane potential, yet compound **1** and **11** even at 50 μM did not display a decrease of MMP. In pilot studies, the toxic effects of compounds **1**, **11** and **18** on LO-2 cells were tested in a CCK8 assay. Supplementary Figure S5 demonstrated that 2.4 μM of compound **18** led to a 50% reduction of cell viability, which corresponded to a 12-fold more potent cytotoxicity than tolcapone. However, compounds **1** and **11** up to 100 μM displayed no significant toxicity with 95% cell survival, while entacapone without liver toxicity served as a negative control. The cell viability and mitochondria membrane potential is an indicator of cell viability^26^. Therefore, these data supported the idea that these novel non-nitrocatechol COMT inhibitors had the potential of being developed with a reduced risk of liver toxicity.

## 4. Conclusion

In summary, our research led to the discovery of novel COMT inhibitors with a chemical scaffold of pentacyclic triterpene, which is distinct from previously reported ones. Using a combination of the inhibition kinetic assay and molecular docking, we tried and explained the inhibition mechanism and the binding site of the pentacyclic triterpene to COMT. Furthermore, oleanic acid and betulinic acid displayed potent COMT inhibition and significantly less toxicity on the mitochondria membrane potential (MMP) of a human normal liver cell line, serving as an ideal lead compound to develop pentacyclic triterpene-type COMT inhibitors. It was possible that these novel COMT inhibitors can provide a starting point for synthetic efforts to generating related derivatives for further preclinical testing and new drugs used for the treatment of Parkinson’s disease, as adjuncts in L-dopa based therapy, or for the treatment of schizophrenia.

## Supplementary Material

Supplemental Material
