# Supplementary material for: Inhibition of catechol-O-methyltransferase by natural pentacyclic triterpenes: structure–activity relationships and kinetic mechanism
Source: J Enzyme Inhib Med Chem. 2021 May 24;36(1):1079–87. doi: 10.1080/14756366.2021.1928112 (PMC8158265; doi:10.1080/14756366.2021.1928112)
Supplement: Supplemental Material [file IENZ_A_1928112_SM4376.pdf]

Supporting Information

Inhibition of catechol-*O*-methyltransferase by natural pentacyclic triterpenes: structure–activity relationships and kinetic mechanism

Fang-Yuan Wang[a,1], Gui-Lin Wei[a,1], Yu-Fan Fan[a], Dong-Fang Zhao[a], Ping Wang [a,*], Li-Wei Zou [a], Ling Yang[a,*]

[a]Institute of Interdisciplinary Integrative Medicine Research, Shanghai University of Traditional Chinese Medicine, Shanghai, 201203, China

[1]The authors contribute equally to this work.

Figure Legends

Scheme S1 Illustration of the fluorescent responsive mechanism of the probe 3-BTD to COMT

Figure S1 Optimization of the reaction time and substrate concentration as well as Michaelis-Menten kinetic analysis of COMT-catalyzed *O*-methylation of 3-BTD

Figure S2 Chemical structures and COMT-inhibitory IC_{50} curves of quercetin and epicatechin

Figure S3 Kinetics study on the inhibition of COMT by compounds **1**, **11**, and **18** with respect to 3-BTD

Figure S4 Three-dimensional structural model of human COMT interacting with compounds **1**, **11**, and **18**

Figure S5 Cytotoxicity assay of tolcapone, entacapone, compounds **1**, **11**, and **18**

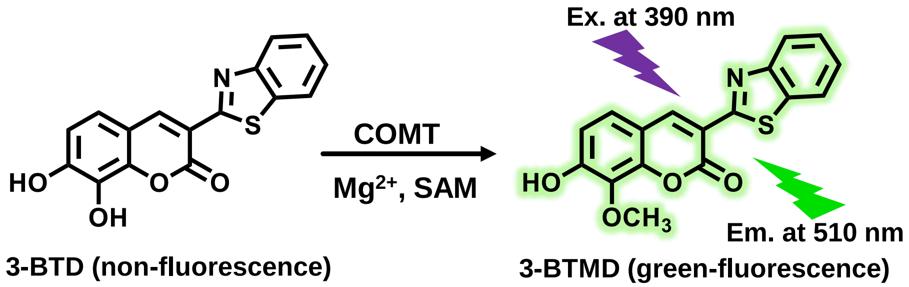

Scheme S1. The fluorescent responsive mechanism of the probe 3-BTD to COMT in the presence of SAM and Mg^{2+} (the wavelengths of excitation and emission set 390 and 510 nm, respectively).

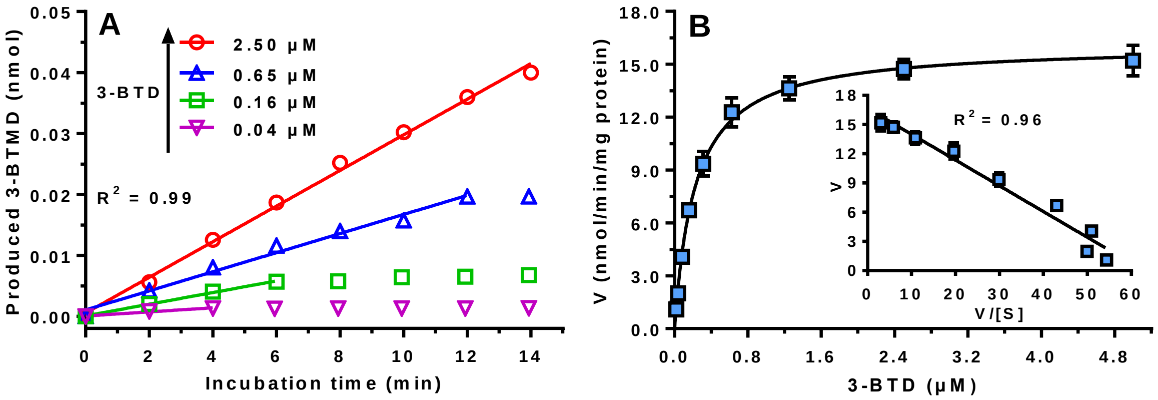

Figure S1 (A) Linear relationship between the incubation time and the formation of the product 3-BTMD that is transformed from 3-BTD of four different concentrations (0.04, 0.16. 0.65 and 2.5 μM). (B) Michaelis-Menten kinetic curve of *O*-methylation of 3-BTD by COMT (the inset show the corresponding Eadie-Hofstee plot). These experiments were performed at 37 °C. The error bars indicate the standard deviation (S.D.) from three independent tests with duplicate determinations.

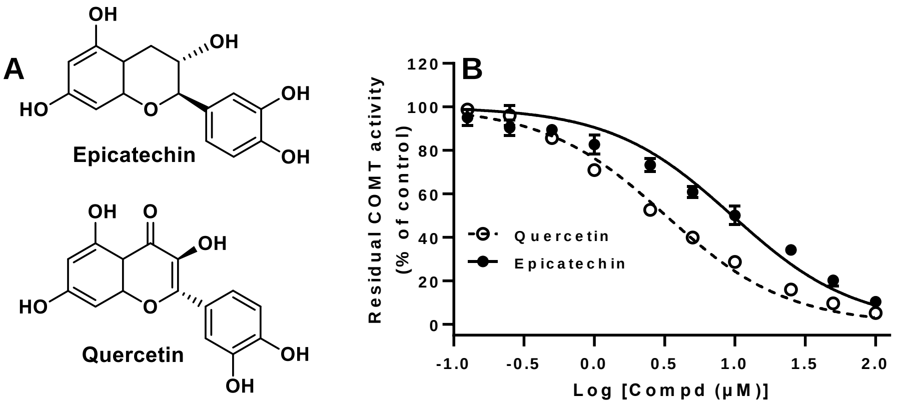

Figure S2 The chemical structures (A) and dose-response curves (B) of epicatechin and quercetin

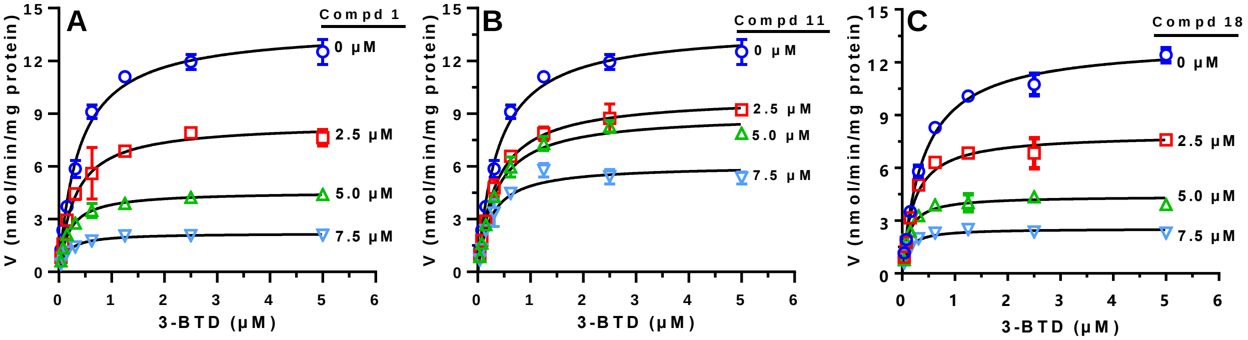

Figure S3 Michaelis-Menten kinetics of 3-BTD in the presence of compounds **1** (A), **11** (B) and **18** (C) of several concentrations. The error bars represent the standard deviation (S.D.) from three independent tests with duplicate determinations.

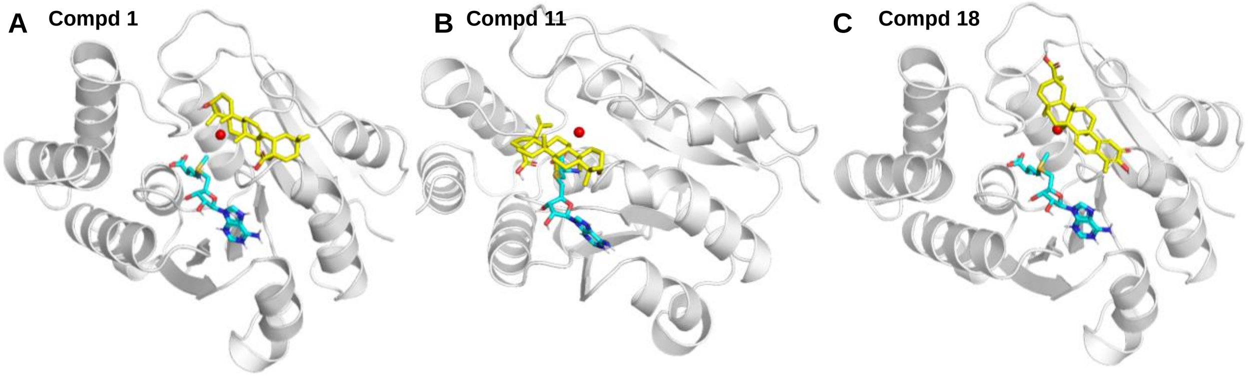

Figure S4 Three-dimensional structural model of human S-COMT interacting with compounds **1**, **11** and **18** (The yellow molecule indicate the tested compound; the red ball represents Mg^{2+}; the blue sky molecule is SAM.)

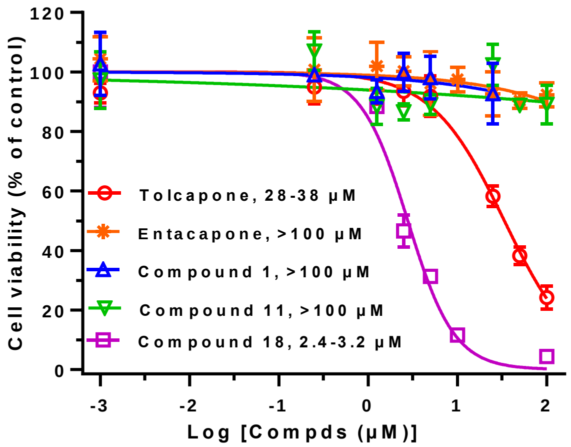

Figure S5 Toxic effects of tolcapone, entacapone, and compounds **1**, **11** and **18** of varying concentrations (from 0.25 to 100 μM) on LO-2 cells. The listed values next to compounds are the concentrations that led to a 50% decrease of cell viability based a DMSO treatment control representing maximum cell viability. The error bars represent the standard deviation (S.D.) from the results of three independent experiments with single determination for this assay.

Table S1. The binding energy values ($\Delta E_{binding}$, kcal/mol) of compounds **1**, **11** and **18** with human S-COMT.

**Compd**	**Chemical name**	**$\Delta E_{binding}$ (kcal/mol)**
**1**	Oleanic acid	-7.1
**11**	Betulinic acid	-6.2
**18**	Celastrol	-7.6
